# Factors associated with compassion fatigue among family caregivers of older adults with dementia

**DOI:** 10.1590/0034-7167-2025-0413

**Published:** 2026-11-06

**Authors:** Mariza Alves Barbosa Teles, Mirna Rossi Barbosa-Medeiros, Antônio Prates Caldeira

**Affiliations:** I Universidade Estadual de Montes Claros. Montes Claros, Minas Gerais, Brazil

**Keywords:** Compassion Fatigue, Caregivers, Family, Aged, Dementia., Desgaste por Empatía, Cuidadores, Familia, Anciano, Demencia.

## Abstract

**Objective:**

to analyze the factors associated with compassion fatigue (CF) among family caregivers of older adults with dementia.

**Methods:**

a cross-sectional study was conducted at a referral center in northern Minas Gerais. The Professional Quality of Life Scale was used to assess CF, seeking associations between its dimensions and sociodemographic, clinical, self-care, and caregiving-related characteristics.

**Results:**

low satisfaction due to compassion was associated with caregivers without a partner and with a negative self-perceived health. Burnout was higher in caregivers without a partner, with a negative self-perceived health, regularly using medication, and who dedicate ≥ 12 hours. Secondary traumatic stress was higher in caregivers without a partner or child, with a negative self-perceived health, regularly using medication, with limited self-care, and with another occupation.

**Conclusion:**

the factors associated with CF are multiple, denote a critical situation, and alert healthcare professionals and managers to the need for differentiated attention to family caregivers.

## RESUMEN

**Objetivo:**

analizar los factores asociados a la fatiga por compasión (FC) en cuidadores familiares de personas mayores con demencia.

**Métodos:**

se realizó un estudio transversal en un centro de referencia en el norte de Minas Gerais. Se utilizó la Professional Quality of Life Scale para evaluar la FC, buscando asociaciones entre sus dimensiones y características sociodemográficas, clínicas, de autocuidado y relacionadas con el cuidado.

**Resultados:**

la baja satisfacción por compasión se asoció con cuidadores sin pareja y con una autopercepción negativa de la salud. El burnout fue mayor en cuidadores sin pareja, con una autopercepción negativa de la salud, en uso regular de medicamentos y que dedicaban ≥ 12 horas. El estrés traumático secundario fue mayor en cuidadores sin pareja ni hijos, con una autopercepción negativa de la salud, en uso regular de medicamentos, con autocuidado limitado y con otra ocupación.

**Conclusión:**

Los factores asociados a la FC son múltiples, indican una situación crítica y alertan a los profesionales y gestores de la salud sobre la necesidad de una atención diferenciada a los cuidadores familiares.

## INTRODUCTION

With the accelerated process of population aging, dementia has become a public health problem worldwide^(1)^. Characterized by impairment of memory, intellect, behavior, and the ability to perform activities of daily living, dementia represents one of the main causes of disability and dependence among older adults, who generally begin to require constant and uninterrupted care in all aspects of daily life^(2,3)^. In this context, families seek to identify a caregiver, whether or not a family member, who, with or without remuneration, provides care to the older adult regarding daily activities^(4)^. In most cases, care is provided by individuals who, due to family ties, take responsibility directly for caring for an ill and/or dependent relative, known as family or informal caregivers^(5)^.

The actions performed by family caregivers comprise a variety of complex activities ranging from assistance with activities of daily living to support in purchasing supplies and accompanying the older adult to healthcare services^(5)^. Family caregivers of older adults frequently experience distress and compassion for the problems faced by the care recipient, which may negatively interfere with their professional quality of life and result in compassion fatigue (CF)^(6)^.

There is no single definition for CF. In general terms, it is considered a form of physical or mental distress whose origin is intrinsically associated with compassion and the act of caring^(7)^. It manifests through behaviors and feelings resulting from the caregiving process or the desire to help a person who is distressed or traumatized, which, in turn, leads the caregiver to a state of biological, psychological, and social exhaustion^(8)^. Under these conditions, care becomes compromised, and both the caregiver and the person receiving care require prompt attention, since this condition prevents full and effective performance^(9)^. Any professionals who deal with the pain and distress of others in their activities are susceptible to CF^(10)^. Within the context of family caregiving, CF emerges when a caregiving relationship grounded in empathy triggers a profound psychological response to stress that progresses to physical, psychological, spiritual, and social exhaustion in the family caregiver^(6)^. The caregiver responds so empathically to the distress of the person under their care that they are unable to detach themselves from the caregiving role, becoming dissatisfied and progressing to CF^(11)^.

The CF construct is characterized by the combination of low levels of compassion satisfaction (CS), high levels of burnout (BO), and secondary traumatic stress (STS). CS represents the condition in which the professional, despite being exposed to negative circumstances, still feels satisfaction and joy in helping people who suffer. BO refers to emotional exhaustion, depersonalization, and reduced personal accomplishment arising in daily work environments characterized by intense pressure, conflicts, and little recognition. STS is a consequence of caring for patients who have experienced distress or are traumatized^(12)^.

In Brazil, studies assessing CF in other contexts were identified, but not among family caregivers, reflecting the gap in knowledge regarding CF among family caregivers^(6,13,14)^. Thus, the following question was formulated: which factors are associated with CF among family caregivers of older adults with dementia? Identifying such factors is highly relevant to support strategies aimed at promoting, preventing, and restoring the health of these caregivers, in addition to highlighting the need to include caregivers within the nursing care pathway.

## OBJECTIVE

To analyze the factors associated with CF among family caregivers of older adults with dementia.

## METHODS

### Ethical aspects

This study was approved by the Research Ethics Committee of *Universidade Estadual de Montes Claros*. All participants were informed about the study objectives and signed the Informed Consent Form.

### Study design, setting, and period

This was a cross-sectional analytical study guided by the recommendations of the STrengthening the Reporting of OBservational studies in Epidemiology, conducted between August and December 2019 at a regional reference center for healthcare for older adults. This is the only specialized public unit for the frail older population in the Northern macro-region of Minas Gerais. The methodological details have been previously published^(15)^.

### Population or sample; inclusion and exclusion criteria

The sample size was calculated based on the annual number of visits by older adults with dementia at the service (in recent years, the average was 5,800 appointments/year), adopting a prevalence of 50%, a 95% confidence level, a 5% sampling error, and 80% power. Considering anticipated losses, the minimum required sample was estimated at 434 caregivers. Selection occurred consecutively: attending physicians identified caregivers who met the inclusion criteria on collection days and referred them to the researchers. Family caregivers of older adults with a medical diagnosis of irreversible dementia, monitored for at least one year, aged ≥18 years, and with at least six months of caregiving experience were included. Caregivers of more than one older adult or those on medical leave at the time of collection were excluded. All approached caregivers agreed to participate in the study, and there were no refusals.

### Study protocol

Data collection was conducted by a trained team during morning and afternoon shifts in the outpatient waiting areas. A structured questionnaire containing sociodemographic, clinical, self-care, and caregiving-related information was used. Completion took approximately 30 minutes. CF was measured using the Professional Quality of Life Scale (ProQOL)^(12)^, whose validity and internal consistency have already been analyzed in Brazil^(10)^. However, until the study by Lynch, Schuster, and Lobo^(16)^, this instrument had been used only for healthcare professionals and was not directed toward family caregivers. Thus, considering the expansion of studies using this instrument with other populations, no specific validity was performed. CF consists of 30 questions divided into ten items for each dimension - CS, BO, and STS - which were the dependent variables of this study. Responses to the ProQOL are presented on a five-item Likert scale, in which “Never” corresponds to “1” and “Very often” corresponds to “5”. The final score for each dimension is interpreted as low if the value is equal to or lower than 22, moderate when the value ranges from 23 to 41, and high when the value is equal to or higher than 42^(12)^. Following methodological recommendations and given the absence of Brazilian norms for caregivers, the three dimensions were categorized by quartiles: low levels of CS for scores below the 25^th^ percentile; and high levels of BO and STS for values above the 75^th^ percentile.

### Analysisof results and statistics

Initially, variables were described using absolute and relative frequencies. Associations between the ProQOL dimensions and independent variables were tested using the chi-square test. Variables with p ≤ 0.20 were included in the multiple binary logistic regression model, remaining in the final model when p < 0.05. Model fit was verified using the Deviance test. Analyses were performed using the Statistical Package for the Social Sciences version 20.0 software.

## RESULTS

The sample consisted of 436 caregivers of older adults with dementia. Most were female (88.1%), aged between 40 and 59 years (55.2%), and were the son/daughter or spouse of the older adult (79.6%), with more than two years of caregiving experience (68.8%). Nearly half of the caregivers dedicated more than eight hours/day to caring for the older adult (49.5%).

Concerning the CF dimensions, CS scores ranged from 23 to 50, BO scores from 10 to 35, and STS scores from 10 to 41. Cut-off points were defined around the 25^th^ percentile (first quartile) for CS and the 75^th^ percentile (fourth quartile) for BO and STS. Thus, low CS was considered for caregivers who scored below 40 points, while high BO and STS were considered for those scoring above 19 and 18 points, respectively. Table 1 presents the bivariate associations between CF dimensions and the sociodemographic characteristics of family caregivers of older adults with dementia.

**Table 1:** Bivariate associations between compassion fatigue dimensions and sociodemographic characteristics among family caregivers of older adults with dementia (N=436). Montes Claros, MG, Brazil, 2019

Variables	Satisfaction					Burnout					Traumatic stress				
	< P25		≥ P25		*p* value	> P75		≤ P75		*p* value	> P75		≤ P75		*p* value
	(n)	(%)	(n)	(%)		(n)	(%)	(n)	(%)		(n)	(%)	(n)	(%)	
**Caregiver sex**					0.253					0.402					0.238
Female	94	24.5	290	75.5		78	20.3	306	79.7		95	24.7	289	75.3	
Male	9	17.3	43	82.7		8	15.4	44	84.6		9	17.3	43	82.7	
**Age (years)**					0.744					**0.003**					**0.066**
≥ 50	43	24.4	133	75.6		47	26.7	129	73.3		50	28.4	126	71.6	
< 50	60	23.1	200	76.9		39	15.0	221	85.0		54	20.8	206	79.2	
**Marital status**					**0.002**					**0.002**					**0.014**
Without a partner	52	31.5	113	68.5		45	27.3	120	72.7		50	30.3	115	69.7	
With a partner	51	18.8	220	81.2		41	15.1	230	84.9		54	19.9	217	80.1	
**Family income**					0.944					**0.011**					**0.015**
≤ 1 minimum wage	37	22.4	128	77.6		40	24.2	125	75.8		49	29.7	116	70.3	
> 1 minimum wage	54	22.1	190	77.9		35	14.3	209	85.7		47	19.3	197	80.7	
**Years of education**					**0.062**					**0.163**					**0.016**
≤ 8	33	19.0	141	81.0		40	23.0	134	77.0		52	29.9	122	70.1	
> 8	70	26.7	192	73.3		46	17.6	216	82.4		52	19.8	210	80.2	
**Relationship to the older adult**					0.337					0.276					**0.043**
Son(daughter)	78	24.8	236	75.2		66	21.0	248	79.0		83	26.4	231	73.6	
Spouse/other	25	20.5	97	79.5		20	16.4	102	83.6		21	17.2	101	82.8	
**Living arrangement**					**0.027**					0.627					0.549
Alone	30	32.3	63	67.7		20	21.5	73	78.5		20	21.5	73	78.5	
Others	73	21.3	270	78.7		66	19.2	277	80.8		84	24.5	259	75.5	

*Note: P25 - 25th Percentile; P75 - 75th Percentile.*

Table 2 shows the bivariate associations between CF dimensions and the clinical and caregiving-related characteristics among family caregivers of older adults with dementia.

**Table 2:** Bivariate associations between compassion fatigue dimensions and clinical and caregiving-related characteristics among family caregivers of older adults with dementia(N=436). Montes Claros, MG, Brazil, 2019

Variables	Satisfaction					Burnout					Traumatic stress				
	< P25		≥ P25		*p* value	> P75		≤ P75		*p* value	> P75		≤ P75		*p* value
	(n)	(%)	(n)	(%)		(n)	(%)	(n)	(%)		(n)	(%)	(n)	(%)	
**Duration of caregiving (years)**					0.212					0.320					**0.041**
≥ 2	76	25.3	224	74.7		63	21.0	237	79.0		80	26.7	220	73.3	
< 2	27	19.9	109	80.1		23	16.9	113	81.1		24	17.6	112	82.4	
**Daily hours dedicated to caregiving**					0.249					**0.002**					**0.004**
≥ 12	18	19.1	76	80.9		29	30.9	65	69.1		33	35.1	61	64.9	
< 12	85	24.9	257	75.1		57	16.7	285	83.3		71	20.8	271	79.2	
**Attended a caregiver training course**					**0.070**					0.504					**0.035**
No	101	24.1	311	76.4		80	19.4	332	80.6		94	22.8	318	77.2	
Yes	2	8.3	22	91.7		6	25.0	18	75.0		10	41.7	14	58.3	
**Sleeps in the same house**					0.237					0.327					**0.103**
Yes	51	21.4	187	78.6		51	21.4	187	78.6		64	26.9	174	73.1	
No	52	26.3	146	73.7		35	17.7	163	82.3		40	20.2	158	79.8	
**Shares caregiving responsibilities**					0.979					0.255					**0.159**
No	32	23.7	103	76.3		31	23.0	104	77.0		38	28.1	97	71.9	
Yes	71	23.6	230	76.4		55	18.3	246	81.7		66	21.9	235	78.1	
**Regular medication use**					0.668					**0.002**					**<0.001**
Yes	47	22.7	160	77.3		54	26.1	153	73.9		66	31.9	141	68.1	
No	56	24.5	173	75.5		32	14.0	197	86.0		38	16.6	191	83.4	
**Leisure hours per day**					0.965					**0.014**					**0.001**
≤ 2 hours	48	23.5	156	76.5		30	14.7	174	85.3		34	16.7	170	83.3	
> 2 hours	55	23.7	177	76.3		56	24.1	176	75.9		70	30.2	162	69.8	
**Dedicates time to self-care**					0.793					**0.001**					**<0.001**
No	18	22.5	62	77.5		26	32.5	54	67.5		38	47.5	42	52.5	
Yes	85	23.9	271	76.1		60	16.9	296	83.1		66	18.5	290	81.5	
**Has another occupation**					0.874					**0.019**					**<0.001**
Yes	56	23.3	184	76.7		57	23.8	183	76.3		80	33.3	160	66.7	
No	47	24.0	149	76.0		29	14.8	167	85.2		24	12.2	17	87.8	
**Self-perceived health**					**0.031**					**<0.001**					**<0.001**
Negative	48	29.3	116	70.7		51	31.1	113	68.9		63	38.4	101	61.6	
Positive	55	20.2	217	79.8		35	12.9	237	87.1		41	15.1	231	84.9	

*Note: P25 - 25th Percentile; P75 - 75th Percentile.*

Variables that presented a significance level up to 20% and were included in the multiple analysis regarding the CS, BO, and STS dimensions are highlighted in Table 2.

Table 3 presents the results of the multiple analysis between the CF scale dimensions and the independent variables in the final model. The variables associated with the outcomes were “marital status”, “self-perceived health”, “regular medication use”, “daily hours dedicated to caregiving”, “relationship to the older adult”, “self-care”, and “having another occupation in addition to caregiving”.

**Table 3:** Multiple analysis of factors associated with compassion fatigue among family caregivers of older adults with dementia (N=436). Montes Claros, MG, Brazil, 2019

Variables/dimensions	OR_b_ (95%CI)	OR_a_ (95%CI)	*p* value
***Lowsatisfaction***			
**Marital status**			0.005
Without a partner	1.98 (1.27-3.10)	1.76 (1.10-2.80)	
With a partner	1.00	1.00	
**Self-perceived health**			0.017
Negative	1.63 (10.4-2.56)	1.76 (1.09-2.79)	
Positive	1.00	1.00	
***Burnout***			
**Marital status**			0.004
Without a partner	3.06 (1.88 - 4.96)	2.26 (1.31-3.89)	
With a partner	1.00	1.00	
**Self-perceived health**			0.007
Negative	2.10 (1.31-3.39)	2.16 (1.24-3.79)	
Positive	1.00	1.00	
**Regular medication use**			0.009
Yes	2.17 (1.34-3.53)	2.16 (1.21-3.86)	
No	1.00	1.00	
**Daily hours dedicated to caregiving**			0.016
≥ 12	2.23 (1.32-3.76)	2.07 (1.15-3.73)	
< 12	1.00	1.00	
***Traumatic stress (fatigue)***			
**Marital status**			0.024
Without a partner	1.75 (1.12 - 2.73)	1.85 (1.08-3.15)	
With a partner	1.00	1.00	
**Relationship to the older adult**			0.015
Son(daughter)	1.73 (1.01-2.94)	2.20 (1.17-4.15)	
Spouse/other	1.00	1.00	
**Self-perceived health**			0.001
Negative	3.51 (2.22-5.55)	2.51 (1.17-4.33)	
Positive	1.00	1.00	
**Regular medication use**			0.007
Yes	2.35 (1.49-3.71)	2.12 (1.22-3.68)	
No	1.00	1.00	
**Dedicates time to self-care**			<0.001
No	3.97 (2.38-6.64)	3.03 (1.66-5.52)	
Yes	1.00	1.00	
**Occupation in addition to caregiving**			0.001
Yes	3.58 (2.16-5.93)	2.56 (1.47-4.44)	
No	1.00	1.00	

*Note: OR - Odds Ratio; CI - Confidence Interval.*

## DISCUSSION

This study enabled the analysis of factors associated with CF among family caregivers of older adults with dementia. This is an innovative approach to a complex construct that, although widely used in the context of formal healthcare workers, is still scarcely applied to family workers, such as family caregivers of older adults^(6,7,9,11,14,16)^. Only one study using the ProQOL scale with family caregivers was identified^(16)^. Other studies involving this population and this topic are qualitative in nature and refer to the experiences of family members caring for a parent in a Nursing Home (NH) or nurses who provided care for their parents with chronic illnesses in institutional settings^(17,18)^. Thus, the analyses performed include studies involving healthcare professionals due to the scarcity of research with caregivers of older adults.

An investigation^(16)^ demonstrated that most participants reported high levels of caregiver burden, but moderate to low levels of CF, indicating that despite the high caregiver burden and moderate CF, family caregivers are able to provide care and find satisfaction in their role. In an Arab study with family caregivers, the authors identified a significant effect of burden on CF, which was found to be high among the 300 assessed families, emphasizing the mediating role of family support^(18)^. Another study highlighted the lack of such support based on caregivers’ statements mentioning the need for a strong sense of responsibility to support their relatives, while simultaneously demonstrating signs of hopelessness, guilt, resentment, and social isolation^(19)^.

A study conducted in Istanbul found exhaustion with repercussions in all areas of life, encompassing relationships with family and friends, professional life, and physical and psychological health^(20)^. Another study identified depression, burden, caregiver strain, and reduced quality of the relationship with the care recipient as consequences of CF. These factors may determine whether children decide to remain caregivers, institutionalize their family member with dementia in an NH, or transfer care responsibilities to others. Furthermore, there is also an increased risk of abuse or neglect^(14)^. Caregivers with CF may also withdraw from caring for their family members^(11)^. Other reactions arising from exhaustion, such as stress, feelings of guilt, and impatience, may also negatively influence daily life and the family context^(21)^.

There is therefore a consensus that family caregivers experience not only work overload, but also substantial difficulty managing their feelings and emotions, leaving them vulnerable to exhaustion in caregiving activities, which leads to CF^(9)^. A systematic review involving family caregivers of older adults with dementia demonstrated that BO negatively affects caregivers’ quality of life and is associated with depressive and anxiety symptoms, as well as abusive behaviors^(22)^.

This study showed that caregivers without a partner were more likely to present low CS scores and high BO and STS scores than those with a partner. A Brazilian study revealed that nurses living with a partner perceived work with greater satisfaction. However, this variable did not remain in the final model^(23)^. The presence of a partner may provide social and family support for the professional’s psychological and emotional structure in the workplace^(24)^. In contrast to the present study, research with healthcare professionals caring for patients with dementia found that widowed and married groups had higher emotional exhaustion and depersonalization scores than single individuals^(20)^. A recent study conducted in Zambia among nurses working in university hospitals revealed that being married was associated with higher STS and BO scores compared to the reference category of being single^(25)^. The observed divergences may be related, at least in part, to differences in the socioeconomic and cultural conditions of each location. Nevertheless, this aspect should be further explored in future studies, especially considering that the population assessed in the present study - family caregivers - has not been a focus of international research on the topic.

In addition to the analysis of marital relationships, the findings regarding burden and CF among healthcare professionals and family caregivers should be interpreted within a broader context, considering the phenomenon of familism characteristic of Latin families and the gaps in the implementation of public policies aimed at supporting family caregivers.

A study related to support policies for dependent older adults, including some European countries and Brazil, revealed that the European countries investigated have invested and continue to invest in a state policy on care, incorporating it into the social security system, and have developed support and protection strategies for both dependent older adults and family caregivers, including financial benefits, training, and respite periods^(26)^.On the other hand, in Brazil, although there are laws protecting older adults, a large proportion of dependent older adults remains almost exclusively under the responsibility of their families. However, this family-centered approach has not been accompanied by an effective national policy to support family caregivers^(26)^. Thus, while formal caregivers have professionally defined activities and are institutionally recognized, family caregivers are often exposed to uninterrupted workloads, which, combined with the absence of public policies, increases the social invisibility of family caregivers and intensifies processes of physical and psychological burden.

In Brazil, there are successful localized municipal experiences that illustrate the possibility of implementing support for family caregivers, such as the Enhanced Care Program (*Programa Maior Cuidado*) in Belo Horizonte, in which dependent older adults and their families living in extreme poverty receive assistance from a formal caregiver on certain days and at specific times^(27)^, and the Older Adult Companion Program (*Programa de Acompanhante ao Idoso*) in São Paulo, composed of professionals trained in geriatrics aimed at providing biopsychosocial home care to clinically and socially vulnerable individuals^(28)^. However, these examples do not constitute a policy. To ensure quality home care, it is necessary to invest in family caregivers by providing them with support in terms of knowledge, practices, and financial assistance^(26)^. Therefore, it is imperative that the protection of dependent older adults be included in the Brazilian policy agenda, overcoming the historical tradition that places responsibility for care exclusively on families^(26)^.

Family caregivers who reported negative self-perceived health were more likely to develop low CS and high BO and STS scores. The results of this study are similar to those of a Brazilian study involving 469 healthcare professionals working with critical/chronic patients, which found low CS levels associated with negative self-perceived health^(29)^. A study involving NH nurses in the United States and Canada reported that professionals who stated that their health was poor were more likely to develop BO and CF^(30)^. These findings are also consistent with another study involving family caregivers, which found that those who rated their health as fair or poor reported more STS than those who characterized it as good or excellent^(16)^. Healthcare professionals who perceived their health status as fair, poor, or very poor were also more likely to develop STS^(29)^. Considering that STS denotes the fear and emotions caregivers experience when assisting or attempting to help a person experiencing distress^(8)^, caregivers with health problems become more vulnerable, as they simultaneously experience negative feelings related both to their own health and to the distress of their relatives^(16)^.

Caregivers who reported regular medication use were more likely to develop BO and STS compared to those who did not use medications. In another study, healthcare professionals who reported using sleep medication were at greater risk of developing BO and STS^(29)^. No other studies assessing this same association specifically among family caregivers of older adults with dementia were identified.

As shown in the findings of this study, family caregivers who dedicated 12 or more hours per day to caring for older adults with dementia were more likely to develop BO. Family caregivers who provided care for more than 25 hours per week presented significantly more BO and STS than those who worked fewer hours^(16)^. An important finding in this study is that it was not the number of years dedicated to caregiving that interfered with CF, but rather the number of weekly hours, suggesting that intensity, rather than duration, is the factor responsible for the observed result.

Another finding of this study was that caregivers who were sons or daughters of the older adult were more likely to develop STS. Similarly to this study, other investigations involving adult daughters caring for parents with dementia demonstrated that attachment, empathic capacity, and caregivers’ concern for their parents may contribute to the development of CF^(11,14)^. Among family caregivers of older adults with dementia, caregiving is almost always perceived as an “obligation”, a responsibility, which in turn makes care more burdensome^(15)^. Adult daughters caring for older parents face the dual task of understanding the distress of the older parent while simultaneously dealing with the demands of daily life, feeling dissatisfied with life but unable to disengage from caregiving responsibilities toward the parent^(11)^.

Participants in this study who reported not dedicating time to self-care and those who had another occupation in addition to caring for the older adult with dementia were more likely to develop STS. No national or international studies addressing these CF dimensions specifically among family caregivers of older adults with dementia were identified. However, one study showed a significant association between family caregiver burden and CF^(18)^. High caregiver burden among family caregivers of older adults with dementia is a finding consistent with previous studies^(15,31)^. Caregiver burden was the strongest predictor of both BO and STS^(16)^.

In a national study, the authors, addressing how informal caregivers of older adults with dementia perceive their own work, conclude that identifying personal needs can raise awareness among healthcare professionals regarding the importance of considering these aspects when proposing caregiver-focused interventions^(32)^. In a systematic review, the authors conclude that care planning for people with dementia must also take into account family caregivers’ needs to promote better well-being and quality of life. Emphasis is placed on the importance of emotional support, the strengthening of resilience, and the need for activities that include regular respite and respect for caregivers’ identity^(33)^.

### Study limitations

As this was a cross-sectional investigation, it is not possible to establish causal inferences between CF and the assessed independent variables. Since the instrument used is not commonly applied to family caregivers, comparison with other studies in the literature is also limited. The selection of the assessed group was performed through the older adult care service, using the schedule of medical appointments, which may represent an important difference compared to caregivers randomly selected in their own homes.

### Contributions to nursing, health, or public policies

This study has the merit of assessing a large sample and addressing aspects that have so far been scarcely explored among family caregivers of older adults with dementia. It presents significant associations that had not previously been evidenced in national and international literature, relating CF dimensions and associated factors. These findings enable nurses to identify and monitor factors influencing CF among these caregivers, as well as implement interventions directed toward identified risk and protective factors. They may also support emotional and psychosocial support programs, health education, stress management, and strategies aimed at promoting and strengthening caregivers’ resilience. Caring for this population group should be a collective responsibility, extending from the family to society as a whole. It is essential for the State to develop integrated public policies that ensure the dignity of older adults and support informal caregivers as central elements in this process.

## CONCLUSIONS

This study demonstrated that there are low levels of CS among family caregivers of people with dementia who do not have a partner and who present negative self-perceived health. In addition, the variables “marital status without a partner”, “negative self-perceived health”, “regular medication use”, and “time dedicated to caregiving ≥ 12 hours per day” were determinants of BO scores. In turn, variables such as “not having a partner”, “being the son/daughter of the older adult”, “having negative self-perceived health”, “regular medication use”, “limited self-care”, and “having another occupation in addition to caregiving” predicted the presence of STS among caregivers. It is concluded that these variables expose family caregivers to CF and that these findings can and should alert healthcare professionals and managers to provide differentiated care to this group, which is almost always invisible to the community and healthcare services themselves. It is imperative to recognize the complexity of caring for older adults with dementia, including special attention to family caregivers who, due to the chronic and unpredictable nature of caregiving, take on accumulating tasks in addition to the psychological distress inherent to caring for a family member.

Although this study allowed for an innovative analysis of the factors associated with CF among family caregivers of older adults with dementia, as this was a cross-sectional investigation, it is not possible to establish causal inferences between CF and the independent variables investigated. Furthermore, due to the scarcity of research involving caregivers in the family context, the analyses performed included studies with healthcare professionals. Therefore, further research is suggested specifically involving family caregivers and, above all, longitudinal studies using CF measurement instruments specifically validated for family caregivers, in order to overcome the knowledge gap regarding CF among these caregivers. Considering the existing gap in the nursing literature on CF among family caregivers, it is expected that this study may guide nurses in implementing interventions focused on the risk and protective factors related to CF among family caregivers of older adults with dementia.

## Data Availability

Research data is only available upon request.
