# Supplementary material for: Factors associated with compassion fatigue among family caregivers of older adults with dementia
Source: Rev Bras Enferm. 2026 Nov 6;79:e20250413. doi: 10.1590/0034-7167-2025-0413 (PMC13743050; doi:10.1590/0034-7167-2025-0413)
Supplement: Translation

# Translation: Fatores associados à fadiga por compaixão entre cuidadores familiares de pessoas idosas com demências

**DOI:** 10.1590/0034-7167-2025-0413pt

**Authors:** Mariza Alves Barbosa Teles, Mirna Rossi Barbosa-Medeiros, Antônio Prates Caldeira

**Affiliations:** I Universidade Estadual de Montes Claros. Montes Claros, Minas Gerais, Brasil

**Keywords:** Fadiga por Compaixão, Cuidadores, Família, Idoso, Demência.

## Abstract

**Objetivo:**

analisar os fatores associados à fadiga por compaixão (FC) entre cuidadores familiares de idosos com demência.

**Métodos:**

estudo transversal, realizado em um centro de referência no norte de Minas Gerais. Utilizou-se a *Professional Quality of Life Scale* para avaliar FC, buscando associação entre suas dimensões e características sociodemográficas, clínicas, de autocuidado e associadas ao cuidado.

**Resultados:**

baixa satisfação por compaixão esteve associada a cuidadores sem companheiro e com autopercepção negativa da saúde. *Burnout* foi maior em cuidadores sem companheiro, com autopercepção negativa de saúde, em uso regular de medicamentos e que se dedicam ≥ 12 horas. Estresse traumático secundário foi maior em cuidadores sem companheiro, filho(a), com autopercepção negativa de saúde, em uso regular de medicamentos, com autocuidado limitado e com outra ocupação.

**Conclusão:**

os fatores associados à FC são múltiplos, denotam uma situação crítica e alertam profissionais e gestores de saúde para uma atenção diferenciada aos cuidadores familiares.

## INTRODUÇÃO

Com o acelerado processo de envelhecimento populacional, a demência se tornou um problema de saúde pública em todo o mundo^(1)^. Caracterizada pelo comprometimento da memória, do intelecto, do comportamento e da capacidade de realizar as atividades de vida diária, a demência representa uma das principais causas de incapacidade e dependência entre os idosos que, geralmente, passam a necessitar de cuidados constantes e ininterruptos em todos os aspectos do cotidiano^(2,3)^. É nesse contexto que as famílias buscam identificar um cuidador, membro ou não da família, que, com ou sem remuneração, realiza o cuidado à pessoa idosa em relação às atividades diárias^(4)^. Na maioria dos casos, os cuidados são prestados por indivíduos que, por possuírem vínculos parentais, responsabilizam-se diretamente pelo ato de cuidar de um familiar doente e/ou dependente, conhecidos como cuidadores familiares ou informais^(5)^.

As ações desenvolvidas pelos cuidadores familiares compreendem uma variedade de atividades complexas que vão desde o cuidado na execução das atividades de vida diárias até o exercício e/ou suporte na compra de mantimentos e acompanhamento da pessoa idosa aos serviços de saúde^(5)^. Cuidadores familiares de idosos, frequentemente, vivenciam o sofrimento e a compaixão pelo problema do seu destinatário dos cuidados, o que pode interferir negativamente em sua qualidade de vida profissional e resultar em fadiga por compaixão (FC)^(6)^.

Não existe uma definição única para FC. Em linhas gerais, é considerada uma forma de sofrimento físico ou mental, cuja origem está intrinsecamente associada à compaixão e ao ato de cuidar^(7)^. Manifesta-se por meio de comportamentos e sentimentos decorrentes do processo de cuidado ou do desejo de ajudar uma pessoa em sofrimento ou traumatizada, o que, por sua vez, leva a pessoa que cuida a um estado de esgotamento biológico, psicológico social^(8)^. Nessas condições, o cuidado fica comprometido, e tanto o cuidador quanto a pessoa a ser cuidada precisam de pronta atenção, já que a condição impossibilita uma atuação plena e efetiva^(9)^. Estão sujeitos à FC quaisquer profissionais que lidam, em suas atividades, com a dor e o sofrimento do outro^(10)^. No âmbito do cuidado familiar, a FC surge quando uma relação de cuidado fundamentada na empatia desencadeia uma resposta psicológica profunda ao estresse que progride para exaustão física, psicológica, espiritual e social no cuidador familiar^(6)^. O cuidador corresponde tão empaticamente ao sofrimento da pessoa que está sob seus cuidados que não consegue se desvincular de sua tarefa de cuidador, sentindo-se insatisfeito e progredindo para FC^(11)^.

O construto FC caracteriza-se pela combinação de baixos níveis de satisfação por compaixão (SC), elevados níveis de *burnout* (BO) e estresse traumático secundário (ETS)^(8)^. A SC representa a condição em que o profissional, apesar de exposto a circunstâncias negativas, ainda sente satisfação e alegria em colaborar com pessoas que sofrem. BO refere-se a uma exaustão emocional, despersonalização e redução da realização pessoal que surge no cotidiano de trabalho determinado por intensas pressões, conflitos e pouco reconhecimento. E ETS é uma consequência do cuidado a pacientes que passaram por algum sofrimento ou que estão traumatizados^(12)^.

No Brasil, foram encontrados estudos que avaliam a FC em outros contextos, mas não entre cuidadores familiares, refletindo a lacuna de conhecimentos sobre FC entre cuidadores familiares^(6,13,14)^. Assim, formulou-se a seguinte pergunta: quais fatores estão associados à FC entre cuidadores familiares de idosos com demências? Identificar tais fatores torna-se bastante relevante para subsidiar estratégias voltadas à promoção, prevenção e recuperação da saúde desses cuidadores, além da necessidade de incluir os cuidadores na linha de cuidados da equipe de enfermagem.

## OBJETIVO

Analisar os fatores associados à FC entre cuidadores familiares de idosos com demência.

## MÉTODOS

### Aspectos éticos

Este estudo foi aprovado pelo Comitê de Ética em Pesquisa da Universidade Estadual de Montes Claros. Todos os participantes foram esclarecidos quanto aos objetivos da pesquisa e assinaram o Termo de Consentimento Livre e Esclarecido.

### Desenho, local e período do estudo

Trata-se de estudo transversal, analítico, norteado pelas recomendações do *STrengthening the Reporting of OBservational studies in Epidemiology*, realizado entre agosto e dezembro de 2019 em um centro de referência regional para a atenção à saúde de pessoas idosas. Essa é a única unidade pública especializada para a população idosa frágil da macrorregião Norte de Minas Gerais. Os detalhes metodológicos já foram previamente publicados^(15)^.

### População ou amostra; critérios de inclusão e exclusão

O tamanho amostral foi calculado a partir do número anual de atendimentos de pessoas idosas com demência no serviço (nos últimos anos, a média foi de 5.800 consultas/ano), adotando-se prevalência de 50%, nível de confiança de 95%, erro amostral de 5% e poder de 80%. Considerando as perdas previstas, estimou-se a necessidade mínima de 434 cuidadores. A seleção ocorreu de forma consecutiva: os médicos assistentes identificavam, nos dias de coleta, os cuidadores que atendiam aos critérios de inclusão e os encaminhavam aos pesquisadores. Incluíram-se cuidadores familiares de pessoas idosas com diagnóstico médico de demência irreversível, acompanhadas há pelo menos um ano, com idade ≥18 anos e pelo menos seis meses de experiência no cuidado. Excluíram-se cuidadores de mais de um idoso ou em licença médica no momento da coleta. Todos os cuidadores abordados consentiram em participar do estudo, não havendo recusas.

### Protocolo do estudo

A coleta foi conduzida por equipe treinada, em turnos matutino e vespertino, nas áreas de espera do ambulatório. Utilizou-se um questionário estruturado com informações sociodemográficas, clínicas, de autocuidado e associadas ao cuidado prestado. O preenchimento durava em média 30 minutos. A mensuração da FC foi conduzida a partir da *Professional Quality of Life Scale* (ProQOL)^(12)^, cuja validade e consistência interna já foram analisadas no Brasil^(10)^. Contudo, até o estudo de Lynch, Schuster e Lobo^(16)^, esse instrumento era utilizado apenas para atender aos profissionais de saúde, não sendo direcionado aos cuidadores familiares. Assim, considerando a expansão de estudos com o referido instrumento para outros públicos, não foi realizada validação específica. A FC é constituída por 30 questões, divididas em dez para cada dimensão- SC, BO e ETS-, sendo estas as variáveis dependentes deste estudo. As respostas ao ProQOL são apresentadas em uma escala de cinco itens do tipo Likert, em que “Nunca” corresponde a “1” e “Muito frequentemente” corresponde a “5”. A pontuação final para cada dimensão é interpretada como: baixa, se o valor for igual ou menor que 22; média, quando o valor estiver entre 23 e 41; e alta, quando o valor for igual ou superior a 42^(12)^. Seguindo recomendações metodológicas e diante da ausência de normas brasileiras para cuidadores, as três dimensões foram categorizadas por quartis: baixos níveis de SC para escores abaixo do percentil 25; e altos níveis de BO e de ETS para valores acima do percentil 75.

### Análise dos resultados e estatística

Inicialmente, as variáveis foram descritas por frequências absolutas e relativas. As associações entre as dimensões do ProQOL e as variáveis independentes foram testadas com qui-quadrado. Variáveis com p ≤ 0,20 foram incluídas em modelo de regressão logística binária múltipla, permanecendo no modelo final aquelas com p < 0,05. O ajuste foi verificado pelo teste de *Deviance*. As análises foram realizadas no *software Statistical Package for the Social Sciences*, versão 20.0.

## RESULTADOS

A amostra foi composta por 436 cuidadores de pessoas idosas com demência. A maioria era do sexo feminino (88,1%), com idade de 40 a 59 anos (55,2%), sendo filho(a) ou cônjuge da pessoa idosa (79,6%) e que atuava na atividade há mais de dois anos (68,8%). Quase a metade dos cuidadores destinava mais de oito horas/dia para cuidar da pessoa idosa (49,5%).

Em relação às dimensões da FC, os escores da dimensão SC variaram de 23 a 50, os do BO, de 10 a 35, e os do ETS, de 10 a 41. Os pontos de corte foram definidos em torno dos percentis 25 (primeiro quartil) para a SC e 75 (quarto quartil) para a BO e a ETS. Dessa forma, foi considerada baixa presença de SC para os cuidadores que apresentaram escores abaixo de 40 pontos, e elevada presença de BO e ETS para aqueles que pontuaram acima dos escores 19 e 18, respectivamente. A Tabela 1 apresenta as associações bivariadas entre as dimensões da FC e os aspectos sociodemográficos dos cuidadores familiares de pessoas idosas com demência.

**Tabela 1:** Associações bivariadas entre as dimensões da fadiga por compaixão e os aspectos sociodemográficos entre cuidadores familiares de pessoas idosas com demência (N=436). Montes Claros, MG, Brasil, 2019

Variáveis	Satisfação					*Burnout*					Estresse traumático				
	< P25		≥ P25		Valor de *p*	> P75		≤ P75		Valor de *p*	> P75		≤ P75		Valor de *p*
	(n)	(%)	(n)	(%)		(n)	(%)	(n)	(%)		(n)	(%)	(n)	(%)	
**Sexo do cuidador**					0,253					0,402					0,238
Feminino	94	24,5	290	75,5		78	20,3	306	79,7		95	24,7	289	75,3	
Masculino	9	17,3	43	82,7		8	15,4	44	84,6		9	17,3	43	82,7	
**Idade (anos)**					0,744					**0,003**					**0,066**
≥ 50	43	24,4	133	75,6		47	26,7	129	73,3		50	28,4	126	71,6	
< 50	60	23,1	200	76,9		39	15,0	221	85,0		54	20,8	206	79,2	
**Situação conjugal**					**0,002**					**0,002**					**0,014**
Sem companheiro(a)	52	31,5	113	68,5		45	27,3	120	72,7		50	30,3	115	69,7	
Com companheiro(a)	51	18,8	220	81,2		41	15,1	230	84,9		54	19,9	217	80,1	
**Renda familiar**					0,944					**0,011**					**0,015**
≤ 1 salário mínimo	37	22,4	128	77,6		40	24,2	125	75,8		49	29,7	116	70,3	
> 1 salário mínimo	54	22,1	190	77,9		35	14,3	209	85,7		47	19,3	197	80,7	
**Anos de estudo**					**0,062**					**0,163**					**0,016**
≤ 8	33	19,0	141	81,0		40	23,0	134	77,0		52	29,9	122	70,1	
> 8	70	26,7	192	73,3		46	17,6	216	82,4		52	19,8	210	80,2	
**Parentesco**					0,337					0,276					**0,043**
Filho(a)	78	24,8	236	75,2		66	21,0	248	79,0		83	26,4	231	73,6	
Esposo(a)/outro(a)	25	20,5	97	79,5		20	16,4	102	83,6		21	17,2	101	82,8	
**Com quem reside**					**0,027**					0,627					0,549
Sozinho	30	32,3	63	67,7		20	21,5	73	78,5		20	21,5	73	78,5	
Outros	73	21,3	270	78,7		66	19,2	277	80,8		84	24,5	259	75,5	

*Nota: P25 - Percentil 25; P75 - Percentil 75.*

A Tabela 2 demonstra as associações bivariadas entre as dimensões da FC e os aspectos clínicos e relacionados ao ato de cuidar entre cuidadores familiares de pessoas idosas com demência.

**Tabela 2:** Associações bivariadas entre as dimensões da fadiga por compaixão e os aspectos clínicos e relacionados ao ato de cuidar de cuidadores familiares de idosos com demência (N=436). Montes Claros, MG, Brasil, 2019

Variáveis	Satisfação					*Burnout*					Estresse traumático				
	< P25		≥ P25		Valor de *p*	> P75		≤ P75		Valor de *p*	> P75		≤ P75		Valor de *p*
	(n)	(%)	(n)	(%)		(n)	(%)	(n)	(%)		(n)	(%)	(n)	(%)	
**Tempo de cuidado (anos)**					0,212					0,320					**0,041**
≥ 2	76	25,3	224	74,7		63	21,0	237	79,0		80	26,7	220	73,3	
< 2	27	19,9	109	80,1		23	16,9	113	81,1		24	17,6	112	82,4	
**Horas diárias dedicadas ao cuidado**					0,249					**0,002**					**0,004**
≥ 12	18	19,1	76	80,9		29	30,9	65	69,1		33	35,1	61	64,9	
< 12	85	24,9	257	75,1		57	16,7	285	83,3		71	20,8	271	79,2	
**Realizou curso de cuidador**					**0,070**					0,504					**0,035**
Não	101	24,1	311	76,4		80	19,4	332	80,6		94	22,8	318	77,2	
Sim	2	8,3	22	91,7		6	25,0	18	75,0		10	41,7	14	58,3	
**Dorme na mesma casa**					0,237					0,327					**0,103**
Sim	51	21,4	187	78,6		51	21,4	187	78,6		64	26,9	174	73,1	
Não	52	26,3	146	73,7		35	17,7	163	82,3		40	20,2	158	79,8	
**Compartilha o cuidado**					0,979					0,255					**0,159**
Não	32	23,7	103	76,3		31	23,0	104	77,0		38	28,1	97	71,9	
Sim	71	23,6	230	76,4		55	18,3	246	81,7		66	21,9	235	78,1	
**Faz uso regular de medicamentos**					0,668					**0,002**					**<0,001**
Sim	47	22,7	160	77,3		54	26,1	153	73,9		66	31,9	141	68,1	
Não	56	24,5	173	75,5		32	14,0	197	86,0		38	16,6	191	83,4	
**Horas lazer por dia**					0,965					**0,014**					**0,001**
≤ 2 horas	48	23,5	156	76,5		30	14,7	174	85,3		34	16,7	170	83,3	
> 2 horas	55	23,7	177	76,3		56	24,1	176	75,9		70	30,2	162	69,8	
**Dedica horas para o autocuidado**					0,793					**0,001**					**<0,001**
Não	18	22,5	62	77,5		26	32,5	54	67,5		38	47,5	42	52,5	
Sim	85	23,9	271	76,1		60	16,9	296	83,1		66	18,5	290	81,5	
**Possui outra ocupação**					0,874					**0,019**					**<0,001**
Sim	56	23,3	184	76,7		57	23,8	183	76,3		80	33,3	160	66,7	
Não	47	24,0	149	76,0		29	14,8	167	85,2		24	12,2	17	87,8	
**Autopercepção de saúde**					**0,031**					**<0,001**					**<0,001**
Negativa	48	29,3	116	70,7		51	31,1	113	68,9		63	38,4	101	61,6	
Positiva	55	20,2	217	79,8		35	12,9	237	87,1		41	15,1	231	84,9	

*Nota:P25 - Percentil 25; P75 - Percentil 75.*

As variáveis que apresentaram nível de significância de até 20% e que foram para a análise múltipla em relação às dimensões SC, BO e ETS estão destacadas na Tabela 2.

A Tabela 3 apresenta o resultado da análise múltipla entre as dimensões da escala de FC e as variáveis independentes no modelo final. As variáveis que se mostraram associadas foram “situação conjugal”, “autopercepção da saúde”, “uso regular de medicamentos”, “horas diárias para o cuidado”, “parentesco com o idoso”, “autocuidado” e “ter outra ocupação além de cuidar”.

**Tabela 3:** Análise múltipla dos fatores associados à fadiga por compaixão entre cuidadores familiares de pessoas idosas com demência (N=436). Montes Claros, MG, Brasil, 2019

Variáveis/dimensões	OR_b_ (IC95%)	OR_a_ (IC95%)	Valor de *p*
***Baixa satisfação***			
**Situação conjugal**			0,005
Sem companheiro(a)	1,98 (1,27-3,10)	1,76 (1,10-2,80)	
Com companheiro(a)	1,00	1,00	
**Autopercepção de saúde**			0,017
Negativa	1,63 (10,4-2,56)	1,76 (1,09-2,79)	
Positiva	1,00	1,00	
***Burnout***			
**Situação conjugal**			0,004
Sem companheiro(a)	3,06 (1,88 - 4,96)	2,26 (1,31-3,89)	
Com companheiro(a)	1,00	1,00	
**Autopercepção de saúde**			0,007
Negativa	2,10 (1,31-3,39)	2,16 (1,24-3,79)	
Positiva	1,00	1,00	
**Uso regular de medicamentos**			0,009
Sim	2,17 (1,34-3,53)	2,16 (1,21-3,86)	
Não	1,00	1,00	
**Horas diárias dedicadas ao cuidado**			0,016
≥ 12	2,23 (1,32-3,76)	2,07 (1,15-3,73)	
< 12	1,00	1,00	
***Estresse traumático (fadiga)***			
**Situação conjugal**			0,024
Sem companheiro(a)	1,75 (1,12 - 2,73)	1,85 (1,08-3,15)	
Com companheiro(a)	1,00	1,00	
**Parentesco**			0,015
Filho(a)	1,73 (1,01-2,94)	2,20 (1,17-4,15)	
Esposo(a)/outro(a)	1,00	1,00	
**Autopercepção de saúde**			0,001
Negativa	3,51 (2,22-5,55)	2,51 (1,17-4,33)	
Positiva	1,00	1,00	
**Uso regular de medicamentos**			0,007
Sim	2,35 (1,49-3,71)	2,12 (1,22-3,68)	
Não	1,00	1,00	
**Dedica horas de autocuidado**			<0,001
Não	3,97 (2,38-6,64)	3,03 (1,66-5,52)	
Sim	1,00	1,00	
**Ocupação além de cuidar**			0,001
Sim	3,58 (2,16-5,93)	2,56 (1,47-4,44)	
Não	1,00	1,00	

*Nota: OR -Odds Ratio; IC - Intervalo de Confiança.*

## DISCUSSÃO

Este estudo permitiu a análise dos fatores associados à FC entre cuidadores familiares de pessoas idosas com demência. Trata-se de uma abordagem inovadora para um construto complexo que, embora seja bastante utilizado no contexto de trabalhadores formais da área da saúde, ainda é pouco utilizado para trabalhadores familiares, como os cuidadores familiares de idosos^(6,7,9,11,14,16)^. Apenas um estudo foi identificado que utilizou a escala ProQOL com cuidadores familiares^(16)^. Outras pesquisas voltadas a esse público e com essa temática são de cunho qualitativo e se referem às vivências de familiares no cuidado a um dos pais em Instituição de Longa Permanência para Idosos (ILPI) ou de enfermeiros que prestaram cuidados a seus pais com doenças crônicas em ambientes institucionais^(17,18)^. Dessa forma, as análises realizadas incluem estudos com profissionais de saúde pela escassez de pesquisas com cuidadores de idosos.

Investigação^(16)^ demostrou que a maioria dos participantes relatou altos níveis de sobrecarga do cuidador, mas níveis moderados a baixos de FC, denotando que apesar da alta sobrecarga do cuidador e da fadiga moderada por compaixão, os cuidadores familiares são capazes de fornecer cuidados e encontrar satisfação na função. Em estudo árabe com cuidadores familiares, os autores identificaram um efeito importante da sobrecarga sobre a FC, que se mostrou elevada entre as 300 famílias avaliadas, enfatizando o papel mediador do apoio familiar^(18)^. Outra pesquisa destaca a falta desse apoio, a partir das expressões dos cuidadores, que mencionam a necessidade de um forte senso de responsabilidade para apoiar seus parentes, demonstrando, ao mesmo tempo, sinais de desesperança, culpa, ressentimento e isolamento social^(19)^.

Pesquisa realizada em Istambul encontrou esgotamento, com repercussões em todas as áreas da vida, abrangendo as relações com a família e amigos, a vida profissional e a saúde física e psicológica^(20)^. Outro estudo encontrou depressão, sobrecarga, tensão do cuidador e uma diminuição da qualidade do relacionamento com o destinatário do cuidado como consequências da FC. Esses fatores podem ser determinantes para os filhos decidirem se permanecem ou não como cuidadores, se levam seu ente com demência para uma ILPI ou se transferem o cuidado para outras pessoas. Além disso, há também risco aumentado de abuso ou negligência^(14)^. Cuidadores com FC podem também renunciar aos cuidados de seus familiares^(11)^. Outras reações advindas do cansaço, como o estresse, o sentimento de culpa e a impaciência, também podem influenciar negativamente o cotidiano e o contexto familiar^(21)^.

Existe, portanto, consenso de que cuidadores familiares vivenciam não apenas uma sobrecarga de trabalho, mas também uma importante dificuldade de manejar seus sentimentos e emoções, deixando-os vulneráveis à exaustão nas atividades, o que leva à FC^(9)^. Revisão sistemática com cuidadores familiares de idosos com demência demonstrou que o BO repercute negativamente na qualidade de vida desses cuidadores, estando associado a sintomas depressivos e ansiosos, bem como a comportamentos abusivos^(22)^.

Este estudo evidenciou que cuidadores sem companheiro apresentaram chance maior de terem baixos escores para SC e altos escores para BO e ETS que aqueles com companheiro. Pesquisa nacional revelou que enfermeiros que vivem com companheiro concebem o trabalho com maior satisfação. Todavia, essa variável não se manteve no modelo final^(23)^. A presença de um companheiro pôde possibilitar apoio social e familiar para a estrutura psíquica e emocional do profissional em seu ambiente de trabalho^(24)^. Em contraposição ao presente estudo, pesquisa com profissionais de saúde que cuidam de pacientes com demência registrou que os grupos de viúvos e casados apresentaram maiores escores de esgotamento emocional e despersonalização do que ser solteiro^(20)^. Estudo recente realizado na Zâmbia entre enfermeiros que trabalham em hospitais universitários revelou que ser casado esteve associado a maiores pontuações de ETS e BO em relação à categoria de referência, que é ser solteiro^(25)^. As divergências observadas podem estar relacionadas, pelo menos em parte, às diferenças das condições socioeconômicas e culturais de cada local. Todavia, esse é um aspecto a ser mais bem explorado em futuras pesquisas, considerando também que o público avaliado no presente estudo, os cuidadores familiares, não têm sido referência nas pesquisas internacionais sobre o tema.

Além da análise de vínculos conjugais, os achados de sobrecarga e de FC entre profissionais de saúde e cuidadores familiares devem ser interpretados em um contexto mais amplo, a partir do fenômeno do familismo característico das famílias latinas e das lacunas na implementação de políticas públicas de suporte ao cuidador familiar.

Estudo relacionado às políticas de apoio aos idosos em situação de dependência, incluindo alguns países europeus e o Brasil, revelou que os países europeus pesquisados investiram e investem em uma política de Estado sobre o cuidado, incorporando-a ao sistema de seguridade social, e desenvolveram estratégias de apoio e proteção tanto ao idoso dependente quanto ao cuidador familiar, incluindo benefícios financeiros, capacitações e períodos de descanso^(26)^. Por outro lado, no Brasil, embora haja leis de proteção à pessoa idosa, grande parte dos idosos dependentes permanece sob a responsabilidade quase que exclusiva das famílias. Contudo, essa centralidade familiar não tem sido acompanhada de uma política nacional efetiva de suporte ao cuidador familiar^(26)^. Dessa forma, enquanto cuidadores formais possuem delimitação profissional de suas atividades e são reconhecidos institucionalmente, os cuidadores familiares frequentemente são expostos a jornadas ininterruptas de trabalho, o que, aliado à ausência de políticas públicas, aumenta a invisibilidade social do cuidador familiar e intensifica processos de sobrecarga física e psíquica.

No Brasil, há experiências municipais localizadas e exitosas que ilustram a possibilidade de implementação de suporte ao cuidador familiar, como a iniciativa Programa Maior Cuidado em Belo Horizonte, em que idosos dependentes e suas famílias, em situação de extrema pobreza, contam com um cuidador formal em alguns dias e horários^(27)^, e o Programa de Acompanhante ao Idoso, em São Paulo, constituído por profissionais com formação em geriatria destinado a oferecer cuidado biopsicossocial em domicílio a pessoas vulneráveis clínica e socialmente^(28)^. Todavia, esses exemplos não constituem uma política. Para um cuidado domiciliar de qualidade, é preciso investir nos cuidadores familiares, fornecendo-lhes suporte no conhecimento, nas práticas e financeiramente^(26)^. Assim, é imperioso que a proteção às pessoas idosas dependentes seja incluída na agenda da política brasileira, superando a tradição histórica de a responsabilidade do cuidado ser exclusivamente das famílias^(26)^.

Cuidadores familiares que informaram autopercepção negativa da saúde apresentaram maior chance de desenvolverem baixa SC e elevados escores de BO e ETS. Os resultados deste estudo se assemelham aos de estudo nacional com 469 profissionais de saúde que trabalham com pacientes críticos/crônicos, o qual encontrou baixos níveis de SC associado à autopercepção negativa da saúde^(29)^. Estudo com enfermeiros de ILPI dos Estados Unidos da América e do Canadá registrou que profissionais que declararam que sua saúde era ruim apresentaram maiores chances de desenvolverem BO e FC^(30)^. Esses resultados são também congruentes com outro estudo que, abordando cuidadores familiares, registrou que aqueles que avaliaram a sua saúde como regular ou má relataram mais ETS do que aqueles que a caracterizaram como boa ou excelente^(16)^. Profissionais de saúde com autopercepção do seu estado de saúde como regular, ruim ou muito ruim também apresentaram maior probabilidade de desenvolverem o ETS^(29)^. Considerando que o ETS denota o medo e as emoções que os cuidadores sentem ao auxiliar ou querer contribuir com uma pessoa em sofrimento^(8)^, quando têm problemas de saúde, tornam-se mais vulneráveis, uma vez que experimentam cumulativamente sentimentos negativos relativamente à sua própria saúde e ao sofrimento dos familiares^(16)^.

Cuidadores que relataram fazer uso regular de medicamentos apresentaram maior chance de desenvolverem BO e ETS, quando comparados aos que não utilizam. Em outro estudo, os profissionais de saúde que relataram fazer uso de medicação para dormir apresentaram maior risco de desenvolvimento de BO e de ETS^(29)^. Não foram identificados outros estudos que avaliaram essa mesma associação, especificamente entre cuidadores familiares de pessoas idosas com demência.

Como apontam os achados deste estudo, cuidadores familiares que dedicavam 12 ou mais horas diárias ao cuidado a idosos com demência apresentaram maiores chances de desenvolverem BO. Cuidadores familiares que prestavam cuidados durante mais de 25 horas por semana apresentaram significativamente mais BO e ETS do que os que trabalhavam menos horas^(16)^. Uma informação relevante neste estudo é que não foi a quantidade de anos dispensado ao cuidado que interferiu na FC, mas o número de horas semanais, sugerindo que é a intensidade, não a duração, o fator responsável pelo resultado encontrado.

Outro achado deste estudo foi que cuidadores que são filhos da pessoa idosa evidenciaram maiores chances de desenvolverem ETS. De forma semelhante a este estudo, outras pesquisas também com filhas adultas cuidadoras de pais com demências demostraram que o apego, a capacidade empática e a preocupação do cuidador com os pais podem contribuir para o desenvolvimento de FC^(11,14)^. Entre cuidadores familiares de idosos com demências, o cuidado é quase sempre percebido como uma “obrigação”, uma responsabilidade, o que, por sua vez, torna o cuidado mais desgastante^(15)^. Filhas adultas cuidadoras lidam com a dupla tarefa de compreender o sofrimento do pai idoso e, ao mesmo tempo, lidar com as demandas do seu cotidiano, sentindo-se insatisfeitas com a vida, mas sem conseguir se desvencilhar do cuidado com o progenitor^(11)^.

Os participantes deste estudo que informaram não dedicar algumas horas para o autocuidado e os que exerciam alguma ocupação, além de cuidar da pessoa idosa com demência, apresentaram maior chance de desenvolver ETS. Não foram encontrados estudos nacionais e nem internacionais que abordam essas dimensões da FC, especificamente entre cuidadores familiares de pessoas idosas com demência. No entanto, pesquisa evidenciou significativa associação da sobrecarga do cuidador familiar na FC^(18)^. Elevada sobrecarga de trabalho entre cuidadores familiares de idosos com demência é um achado consistente com estudos anteriores^(15,31)^. A sobrecarga do cuidador foi o maior preditor tanto de BO quanto de ETS^(16)^.

Em estudo nacional, os autores, abordando a percepção de cuidadores informais de idosos com demência sobre o próprio trabalho, concluem que a identificação de demandas pessoais pode sensibilizar e conscientizar os profissionais de saúde sobre a importância de considerar esses aspectos na proposição de intervenções focadas nos cuidadores^(32)^. Em revisão sistemática, os autores concluem que o planejamento de cuidados às pessoas com demência também deve levar em conta as necessidades do cuidador familiar para promover melhor bem-estar e qualidade de vida. Destaca-se a importância do apoio emocional, do fortalecimento da resiliência e da necessidade de atividades que incluam descanso regular e respeito à identidade do cuidador^(33)^.

### Limitações do estudo

Por ser uma investigação transversal, não se é possível fazer inferências causais entre a FC e as variáveis independentes avaliadas. Pelo fato de o instrumento utilizado não ser habitualmente empregado para cuidadores familiares, a comparação com outros estudos na literatura também é limitada. A seleção do grupo avaliado foi realizada a partir do serviço de atenção à pessoa idosa, aproveitando a agenda das consultas médicas, o que pode representar uma diferença importante em relação a cuidadores aleatoriamente alocados em seus próprios domicílios.

### Contribuições para a área da enfermagem, saúde ou políticas públicas

Este estudo tem o mérito de avaliar uma grande amostra e abordar aspectos até então pouco explorados entre cuidadores familiares de idosos com demência. Ele apresenta associações significativas que não haviam sido evidenciadas em outros trabalhos da literatura nacional e internacional, relacionando as dimensões da FC e os fatores associados a ela. Esses achados permitem ao enfermeiro conhecer e monitorar os fatores que influenciam a FC entre esses cuidadores, bem como implementar intervenções direcionadas aos fatores de risco e protetivos que forem identificados. Podem ainda subsidiar programas de suporte emocional, psicossocial, educação em saúde, gestão do estresse e estratégias que promovam e fortaleçam a resiliência desses cuidadores. O cuidado a esse grupo populacional deve ser uma responsabilidade coletiva, estendendo-se da família para toda a sociedade. É essencial que o Estado crie políticas públicas integradas que garantam a dignidade do idoso e apoiem o cuidador informal como elemento central nesse processo.

## CONCLUSÕES

Este estudo permitiu verificar que existem baixos níveis de SC entre cuidadores familiares de pessoas com demência sem companheiro e com autopercepção negativa da saúde. Ainda, as variáveis “situação conjugal sem companheiro”, “autopercepção negativa de saúde”, “uso regular de medicamentos” e “tempo de cuidado dedicado ao cuidado ≥ 12 horas por dia” foram definidoras dos escores de BO. Por sua vez, variáveis como “não ter companheiro”, “ser filho(a) da pessoa idosa”, “ter autopercepção negativa de saúde”, “fazer uso de regular de medicamentos”, “ter autocuidado limitado” e “ter outra ocupação além de cuidador” foram preditoras de o cuidador apresentar ETS. Conclui-se que tais variáveis expõem os cuidadores familiares à FC e que esses resultados podem e devem alertar profissionais de saúde e gestores para uma atenção diferenciada a esse grupo, quase sempre invisível aos olhos da comunidade e dos próprios serviços de saúde. É imperioso reconhecer a complexidade do cuidado voltado às pessoas idosas com demência, incluindo-se uma atenção especial aos cuidadores familiares, que, pelo caráter crônico e imprevisível do cuidado, assumem tarefas que se acumulam e se somam ao sofrimento psíquico inerente ao cuidar de um ente familiar.

Embora este estudo tenha permitido uma análise inovadora dos fatores associados à FC entre cuidadores familiares de pessoas idosas com demência, por se tratar de uma investigação transversal, não é possível fazer inferências causais entre a FC e as variáveis independentes investigadas. Além disso, em virtude da escassez de pesquisas com cuidadores no contexto familiar, as análises realizadas incluíram estudos com profissionais de saúde. Portanto, sugere-se a realização de mais pesquisas que incluam, especificamente, cuidadores familiares e, sobretudo estudos longitudinais que utilizem instrumentos de medição da FC com validação específica para os cuidadores familiares, a fim de suplantar a lacuna de conhecimentos sobre FC entre esses cuidadores. Considerando o hiato existente na literatura sobre a FC em cuidadores familiares na enfermagem, espera-se que este estudo possa direcionar o profissional enfermeiro na implementação de intervenções voltadas aos fatores de risco e de proteção da FC entre cuidadores familiares de pessoas idosas com demência.

## Data Availability

Os dados de pesquisa só estão disponíveis mediante solicitação.
